# Multi-species genetic connectivity in a terrestrial habitat network

**DOI:** 10.1186/s40462-017-0112-2

**Published:** 2017-10-06

**Authors:** Robby R. Marrotte, Jeff Bowman, Michael G.C. Brown, Chad Cordes, Kimberley Y. Morris, Melanie B. Prentice, Paul J. Wilson

**Affiliations:** 1 Environmental and Life Sciences Graduate Program, Trent University, Peterborough, Canada; 2 Wildlife Research and Monitoring Section, Ontario Ministry of Natural Resources and Forestry, Peterborough, Canada; 3 Biology Department, Trent University, Peterborough, Canada

**Keywords:** Landscape context, Landscape fragmentation hypothesis, Circuitscape, Multi-species connectivity, Pinch point

## Abstract

**Background:**

Habitat fragmentation reduces genetic connectivity for multiple species, yet conservation efforts tend to rely heavily on single-species connectivity estimates to inform land-use planning. Such conservation activities may benefit from multi-species connectivity estimates, which provide a simple and practical means to mitigate the effects of habitat fragmentation for a larger number of species. To test the validity of a multi-species connectivity model, we used neutral microsatellite genetic datasets of Canada lynx (*Lynx canadensis*), American marten (*Martes americana*), fisher (*Pekania pennanti*), and southern flying squirrel (*Glaucomys volans*) to evaluate multi-species genetic connectivity across Ontario, Canada.

**Results:**

We used linear models to compare node-based estimates of genetic connectivity for each species to point-based estimates of landscape connectivity (current density) derived from circuit theory. To our knowledge, we are the first to evaluate current density as a measure of genetic connectivity. Our results depended on landscape context: habitat amount was more important than current density in explaining multi-species genetic connectivity in the northern part of our study area, where habitat was abundant and fragmentation was low. In the south however, where fragmentation was prevalent, genetic connectivity was correlated with current density. Contrary to our expectations however, locations with a high probability of movement as reflected by high current density were negatively associated with gene flow. Subsequent analyses of circuit theory outputs showed that high current density was also associated with high effective resistance, underscoring that the presence of pinch points is not necessarily indicative of gene flow.

**Conclusions:**

Overall, our study appears to provide support for the hypothesis that landscape pattern is important when habitat amount is low. We also conclude that while current density is proportional to the probability of movement per unit area, this does not imply increased gene flow, since high current density tends to be a result of neighbouring pixels with high cost of movement (e.g., low habitat amount). In other words, pinch points with high current density appear to constrict gene flow.

**Electronic supplementary material:**

The online version of this article (10.1186/s40462-017-0112-2) contains supplementary material, which is available to authorized users.

## Background

Much recent thinking about maintaining biodiversity in the face of environmental change suggests that ensuring adequate landscape connectivity is important. Indeed, managing connectivity is considered by some to be one of the key strategies for creating resilient landscapes and adapting to climate change [1]. Landscape connectivity is defined as the degree to which the landscape facilitates or impedes movement among resource patches and is generally considered to be a species-specific trait of landscapes that emerges as a product of landscape structure and species behaviour [2]. This is often referred to as functional connectivity. Landscape connectivity is an explicit management goal for many jurisdictions that have natural heritage or land use plans with connectivity targets [3, 4].

Given the species-specific nature of the landscape connectivity concept, it is not surprising that many studies evaluate single-species landscape connectivity. For example, single-species connectivity estimates have been used as a means to assess suitable habitat for Eurasian lynx (*Lynx lynx*), evaluate the effectiveness of vaccination barrier strategies to control the transmission of rabies virus, and identify the appropriate placement of road mitigation measures to facilitate wildlife crossings in jaguars (*Panthera onca)* [5–7]. Single-species connectivity can be a challenge however, for land use planning, when multiple species are of conservation interest. As such, multi-species connectivity maps have been of interest in recent years [8, 9]. There are generally two different approaches identified for developing multi-species connectivity maps. First, a series of single-species maps can be developed, and then overlaid to produce a consensus map [10]. This approach may be impractical for large-scale conservation however, when there are numerous species of conservation interest. Alternatively, a single, multi-species map can be produced, and attempts made to validate this map to establish its extent of generality [11]. Such multi-species connectivity maps are an attractive option for managers, since they have the benefit of simplicity and practicality.

Functional connectivity implies that animals are successfully moving through landscape elements and functioning as effective members of the local population via successful reproduction and gene flow [12]. Thus, many have argued that gene flow can be used to directly measure such effective movement [13], and numerous examples exist where gene flow is evaluated as a measure of functional connectivity [14–17]. Gene flow has been used in natural heritage or land use planning, and where multi-species connectivity is a goal, a logical consequence is that habitat networks should provide multi-species genetic connectivity [18]. The concept of multi-species genetic connectivity is relatively new, but has been used recently to assess coral reef networks [19, 20]. We are aware of only a few studies where this has been assessed in terrestrial environments. For example, Mech and Hallett (2001) used gene flow to evaluate the effectiveness of corridors for red-backed voles (*Myodes gapperi*) and deer mice (*Peromyscus maniculatus*) [21], and more recently Wultsch et al. (2016) compared effects of habitat fragmentation on gene flow of three large cat species in Belize [22]. In contrast to the field of genetics, multi-species connectivity has been addressed in several landscape ecology studies [23–25].

One challenge in evaluating how landscape structure affects gene flow arises due to the pairwise nature of gene flow estimates (e.g., F_ST_). Similarly, measures of landscape connectivity may also be pairwise. For example, the most popular methods in recent years for measuring connectivity of landscapes are least cost paths [26] and circuit theory [27, 28], both of which provide pairwise measures [29], which quantify the degree of connectivity or isolation between two locations. Traditional statistical tests used to evaluate pairwise genetic distances have come under recent scrutiny due to the potential for spurious correlations [30]. Mantel and partial Mantel tests have been used to link multivariate landscape and genetic data, by measuring correlations between pairwise genetic distances and corresponding geographical distances. These tests may have low power [30, 31] and high error rates [31–34] however, suggesting benefits of analytical approaches that avoid pairwise data.

Recently, Koen et al. [35] evaluated several at-site measures of population-level gene flow. At-site measures differ from pairwise measures in that estimates rely on properties of each sampled location, rather than on pairs of locations. Koen et al. [35] employed a network-based analysis, whereby nodes and edges were site-specific samples and genetic distances between samples, respectively. A simulation study and an empirical validation demonstrated that measures of network edge weight could accurately describe gene flow at nodes due to the settlement phase of dispersal, where settlement is the establishment of residency at a location following a breeding or natal dispersal [35]. Thus, node-based measures could be used as point estimates of genetic connectivity, and compared to point estimates of landscape structure; an approach that would not rely on pairwise data analysis. Point estimates of landscape structure have typically included empirical measures of habitat amount and fragmentation estimated in neighbourhoods of various radii [36–38]. We propose to evaluate an alternative measure of landscape connectivity – a point-based summation of current density, estimated via circuit theory. To our knowledge, current density has not been used before as a measure of genetic connectivity.

Least cost path analysis (LCP) involves estimating the optimal, lowest cost route between source and destination nodes [26]. This measure has been widely used, and shown to effectively measure connectivity in some situations [15, 39]. However, a drawback of LCP is that it assumes that animals have knowledge of the optimal route, which may not be a valid assumption in some cases, especially in the face of a process such as gene flow, which may be widespread. An alternative approach has more recently been proposed using circuit theory that allows the estimation of multiple paths [27]. Circuit theory takes advantage of the analogy between random walks and electricity travelling on a circuit [40] to depict movement probability. Numerous applications of this approach are also documented in the literature (e.g., [41–43]).

There are three key features of circuit theory important to highlight for our purposes. First, one output of circuit theory is called current density, which is spatially referenced, and is proportional to the probability of movement by a random walker [28, 40]. Current density is not a pairwise measure. Rather, it can be sampled at a point, and as such, is distinct from the other common circuit theory measure, resistance distance (or effective resistance), which is a pairwise measure of isolation. Second, current density maps depict emergent effects that exist apart from simple measures of habitat amount. For example, geometric pinch points stand out as areas of high movement probability regardless of underlying habitat conditions [27]. Finally, circuit theory estimates can be systematically undertaken in an omnidirectional manner [11], such that unbiased point samples of current density can accurately estimate local movement probability.

Our main objective was to compare two node-based estimates of connectivity. We compared a measure of multi-species genetic connectivity to another measure derived from omnidirectional current density maps. We used a previously developed terrestrial multi-species connectivity map developed from circuit theory for natural heritage planning in Ontario, Canada [11, 44]. Koen et al. [11] initially developed the methodology for this map, and used fisher (*Pekania pennanti*) telemetry and herpetofaunal roadkill data to validate the results for a study area in eastern Ontario. The methods were subsequently applied by Bowman and Cordes [44] across the full province of Ontario. We sought to evaluate this independently-derived, Ontario connectivity map using multi-species genetic data to test the hypothesis that areas of high connectivity are multi-species gene flow hotspots. We used neutral microsatellite genetic datasets for fisher, American marten (*Martes americana*), southern flying squirrel (*Glaucomys volans*), and Canada lynx (*Lynx canadensis*) sampled in Ontario to test the hypothesis. We also compared the current density map and genetic connectivity estimates to a more traditional measure of landscape structure based on buffered estimates of habitat amount.

## Methods

**Fig. 1:**
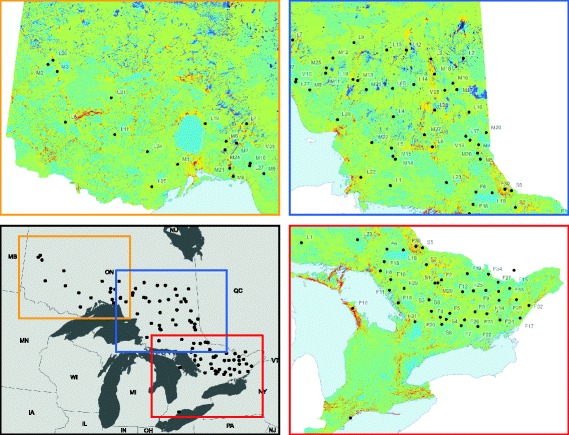
Sampled node locations spatially overlayed on the current density surface. Node IDs are species specific, where F, L, M, S represent Fisher (*Pekania pennanti)*, Canada Lynx (*Lynx canadensis)*, American Marten (*Martes americana)*, and Southern Flying Squirrel (*Glaucomys volans*) respectivly. Current density values have been standardized to a mean of zero, Red to blue values indicate high to low current density, respectively. The study area ranged between about 42.3°N (S7) and 51.3°N (L20)

We used existing population genetic datasets of four terrestrial mammal species to model multi-species genetic connectivity across Ontario, Canada (Fig. 1). These datasets included neutral microsatellite profiles from 702 Canada lynx at 14 loci [11], 653 American marten at 12 loci [41], 657 fisher at 16 loci [45, 46], and 278 southern flying squirrels at 7 loci [47, 48]. From these datasets, we removed individuals that had more than 35% missing alleles. We selected these four species as they were all terrestrial mammals occurring in forested habitats of Ontario for which we had genetic profiles. Our objective was to test whether a multi-species connectivity map could predict the genetic connectivity of all four species despite species-level differences in movement behaviours.

Samples were collected across the Ontario study area between about 42.3°N and 51.3°N. The southern portion of the study area was dominated by agriculture and urban development. Forests in this region represented a transition between southern temperate forests and boreal forests. The northern end of the study area was boreal forest, with a much lower human population density, lower agricultural intensity and little urban development. Fisher and flying squirrel samples were concentrated in the southern portion of the study area, whereas marten and lynx samples were concentrated in northern Ontario (Fig. 1).

We constructed population graphs [49] from the neutral genetic datasets to model and visualize genetic connectivity between sites for each species. This was accomplished by using the software packages *gstudio* and *popgraph* [50, 51] in R (version 3.2.4) to construct networks of connectivity among sample sites representing different populations. All species were sampled using a lattice intended to evaluate population-based network structure (e.g., [45]). In each case, populations were sampled at multiple locations, separated by distances that exceeded typical daily movements for the species in question. Sampled sites were considered network nodes, and the connections between pairs of sites were considered network edges. Networks can be illustrated by a series of nodes oriented in multidimensional space that represent the allelic diversity from that site. Each node is connected by a series of edges representing genetic similarity between nodes; in a saturated network all nodes are connected by edges. Each edge length is proportional to the multivariate genetic covariance between nodes. Within a saturated network, some edges do not adequately describe the overall among-population genetic covariance structure, so we pruned edges that did not compromise the fit of the population graph model to the marker-based population genetic data as described by Dyer and Nason [49]. The shortest path length between nodes along the pruned network is referred to as conditional genetic distance (cGD) [52].

We used a previously published current density map for Ontario that was derived from a circuit theory approach to model omnidirectional, multispecies connectivity based on landscape resistance [11, 44]. The current density map was constructed using Circuitscape v4.0 (www.circuitscape.org) which models a rasterized landscape as a circuit board where each pixel represents resistance to wildlife movement. Expert opinion was used to assign land cover to one of three resistance values as outlined by Koen et al. [11] and at a resolution of 100 m. Circuitscape was used to simulate electrical current flow between source and destination points, where the resulting current density in a pixel is proportional to the probability of animal movement. Importantly, source and destination points for the circuit theory analysis were independent of nodes sampled for our genetic networks. Instead, node pairs were randomly placed outside of the perimeter of the study area, according to the procedure of Koen et al. [11]. The placement of nodes outside of the study area removes the build-up of current near nodes within the study area that has been referred to as ‘node placement bias’ [11], and the random placement of nodes allows the creation of an omnidirectional map. Therefore, we used a model of omnidirectional, multi-species movement probability across a heterogeneous landscape grid of varying permeability. For more details and description of the methodology see Bowman and Cordes [44] and Koen et al. [11]. The expert-derived cost surface had 3 levels of permeability, where 10 was the lowest cost including most types of natural cover, 100 was semi-permeable cover types, and 1000 was the highest cost, including mostly anthropogenic and urbanized land cover types (see Additional file 1: Appendix 1). A novelty of our approach is the use of a point-based sampling method to estimate current density at buffered sample locations via circuit theory. We were able to take this approach because Bowman and Cordes [44] systematically assessed current density across Ontario using the omnidirectional methodology of Koen et al. [11]. Therefore, a point sample of current density should be a meaningful estimate of animal movement and gene flow.

We calculated our measure of node-based genetic connectivity using the average inverse edge weight of cGD for each sampled node, which has been shown to be positively correlated to genetic connectivity [35]. We standardized this measure among species by calculating z-scores, thereby placing the measure of genetic connectivity for each species on the same scale. This made it possible to exclude species as an additional covariate in our models. We compiled the mean and standard deviation of current density at 3 different geographic extents by buffering each node and extracting these values at radii of 6, 20 and 120 km to cover a range of distances relevant to the species being studied. Several of the covariates (cost and current density at different extents) were highly correlated (Additional file 1: Appendix 2); consequently we did a principal component analysis (PCA) and used the first 4 principal components (PCs) that accounted for ~85% of the total variance. We then used the loadings of our covariates to interpret our subsequent models. We accounted for spatial dependency by including spatial coordinates of sampled nodes as additional independent variables and we also accounted for sample size using the number of individuals genotyped at each node. We wanted to know the standardized effect of each of our covariates; therefore we also standardized these values using their z-scores. We carried out our modelling over three different areas. We first modelled the entire region, encompassing all of the sampled nodes of all four species. We then separately modelled the north and the south, including only the species with northern (Canada lynx and American marten) and southern (fisher and southern flying squirrel) distributions in the respective models. We used ordinary least-squares regression to model standardized average inverse edge weight against longitude, latitude, sample size, and our four PCs representing functional connectivity.

## Results

**Fig. 2:**
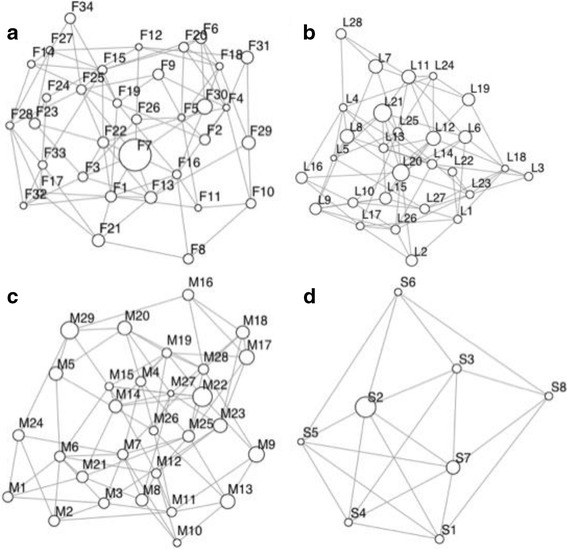
Population graphs representing the genetic relationships among 34 sites of fisher (*Pekania pennanti*, **a**), 28 sites of Canada lynx (*Lynx canadensis*, **b**), 29 sites of American marten (*Martes americana*, **c**), and 8 sites of southern flying squirrels (*Glaucomys volans*, **d**) in Ontario, Canada. Node size is proportional to node centrality and edge length is proportional to the genetic distance between populations

Our population graphs indicated that there was relatively high connectivity within each of the mammal species considered, given the generally well connected graph topologies (Fig. 2). Sample sizes ranged from 6 to 29, 11-47, and 13-39 per node for fisher, marten and lynx, respectively, with flying squirrel showing the greatest variability in sample size with between 6 and 120 samples per node (Additional file 1: Appendix 3). The number of nodes was similar among 3 species, with 34, 29 and 28 nodes for fisher, marten and lynx, whereas flying squirrel had only 8 nodes. Average inverse edge weight was similar between marten (0.284; range: 0.191-0.356) and lynx (0.259; range: 0.182-0.323), which were both higher than fisher (0.191; range: 0.100-0.249) and flying squirrel (0.196; range: 0.137-0.246) (Table 1).

**Table 1:** Average edge weight, average inverse edge weight, mean cost and mean current density of nodes for four terrestrial mammal species sampled throughout Ontario, Canada

Variable	Fisher	Canada Lynx	American Marten	Southern Flying Squirrel
Number of Nodes	34	28	29	8
Average edge weight (range)	5.694 (4.095 – 10.130)	3.972 (3.115 – 5.512)	3.654 (2.814 – 5.276)	5.418 (4.140 – 7.320)
Average inverse edge weight (range)	0.191 (0.100 – 0.249)	0.259 (0.182 – 0.323)	0.284 (0.191 – 0.356)	0.196 (0.137 – 0.246)
6 km buffer mean cost (range)	172.0 (17.3 – 809.9)	83.3 (14.3 – 268.2)	94.1 (13.5 – 283.3)	265.1 (81.4 – 525.4)
20 km buffer mean cost (range)	151.7 (62.8 – 342.1)	97.2 (28.4 – 278.3)	119.8 (24.9 – 282.4)	187.0 (102.1 – 243.7)
120 km buffer mean cost (range)	171.6 (115.3 – 251.2)	130.0 (45.9 – 325.9)	127.4 (62.9 – 211.7)	173.2 (146.8 – 188.5)
6 km buffer mean current (range)	0.100 (−1.080 – 1.579)	0.060 (−1.124 – 0.884)	0.0717 (−0.716 – 1.123)	0.236 (−0.818 – 0.849)
20 km buffer mean current (range)	0.096 (−0.568 – 1.697)	0.057 (−0.663 – 0.684)	0.007 (−0.405 – 0.567)	0.272 (−0.193 – 0.713)
120 km buffer mean current (range)	0.006 (−0.344 – 1.276)	−0.010 (−0.326 – 0.249)	−0.057 (−0.244 – 0.207)	0.156 (−0.047 – 0.329)

We found differing trends with respect to the relationship between buffer size and mean cost estimated for each species, which resulted from both the distribution of each species and the strategy taken for collecting DNA samples. Specifically, for the more northern distributed species (lynx and marten), mean cost increased with increasing buffer size. Alternatively, for the southern distributed species (fisher and southern flying squirrel), mean cost generally decreased as buffer size increased. Specifically, for southern flying squirrel, there was a consistent decline in mean cost as the buffer size increased from 6 km to 20 km to 120 km (Table 1). For fisher, however, the mean cost initially decreased from 6 km to 20 km, but then returned to the same approximate mean cost at a 120 km neighbourhood size (Table 1). Mean current density also varied between buffer sizes for each of our species. For fisher, marten and lynx, mean current density declined with increasing buffer size. For flying squirrel, on the other hand, there was an initial decrease in mean current density from the 6 km to 20 km buffer, but then the current density increased again in the 120 km buffer calculation.

The first four PCs in our PCA analysis explained 36.4%, 28.4%, 13.1%, and 7.2% of the variation, respectively (85.1% cumulative variance explained). PC1 had a negative loading of the mean cost and its standard deviation at all three spatial extents (6 km, 20 km and 120 km) (Table 2). Conversely, PC2 had a positive loading of the mean current and its standard deviation at each spatial scale. PC3 had a combination of negative loading of mean cost with its standard deviation at the broadest extent (120 km) and positive loading of the standard deviation of cost at the finest extent (6 km). The PC4 plane was partitioned by a combination of a positive loading of current at the 20 km extent and a negative loading of the standard deviation of cost at 6 km.

**Table 2:** Principal component (PC) loadings for variables derived from a cost surface and a current density map

Variable Description^a^	PC1^b^	PC2	PC3	PC4
Mean Cost (6 km)	**−0.360**	−0.115	0.261	**−0.421**
Mean Cost (20 km)	**−0.424**	−0.105	−0.013	0.306
Mean Cost (120 km)	**−0.322**	−0.096	**−0.530**	−0.136
Standard Deviation of Cost (6 km)	**−0.380**	−0.044	**0.311**	−0.341
Standard Deviation of Cost (20 km)	**−0.426**	−0.095	−0.017	0.333
Standard Deviation of Cost (120 km)	**−0.332**	−0.096	**−0.527**	−0.077
Mean Current (6 km)	0.075	**0.418**	−0.270	0.260
Mean Current (20 km)	0.012	**0.474**	−0.154	−0.250
Mean Current (120 km)	−0.079	**0.432**	−0.035	−0.364
Standard Deviation of Current (6 km)	−0.242	**0.282**	**0.366**	0.104
Standard Deviation of Current (20 km)	−0.250	**0.347**	0.196	**0.442**
Standard Deviation of Current (120 km)	−0.132	**0.401**	−0.076	−0.090

^a^Numbers in parenthesis indicate the diameter of a buffer over which estimates were calculated

^b^Values with bold font indicate the major contributors of the principal component

In our regression model of the full study area (F = 3.07, df = 7, 89, *P* = 0.006, adjusted R^2^ = 0.131), the only predictor of genetic connectivity (estimated as average inverse edge weight) whose confidence intervals did not overlap 0 was sample size (Table 3). The northern model that included both Canada lynx and American marten had an R^2^ of 0.404 (F = 6.42, df = 7, 49, *P* < 0.0001, adjusted R^2^ = 0.404), whereas the southern model, including fisher and southern flying squirrel, explained less variation (F = 2.84, df = 7, 32, *P* = 0.02, adjusted R^2^ = 0.249). In the northern model, sample size was once again an important predictor of genetic connectivity, as was PC3, which showed a negative effect of cost on genetic connectivity. In contrast, PC2 (related to current density) was an important predictor of genetic connectivity in the southern model (including fisher and southern flying squirrel), as was sample size. The direction of the relationship in the southern model suggested that current density was negatively related to gene flow (Tables 2 and 3).

**Table 3:** Slope (SE) estimates for regression models comparing a node-based measure of genetic connectivity to seven predictor variables

Variable	Full Model	Northern Model	Southern Model
Intercept	1.286 × 10^−16^ (0.093)	1.111 × 10^−16^ (0.101)	7.891 × 10^−17^ (0.135)
X	0.208 (0.207)	−0.228 (0.198)	0.046 (0.172)
Y	0.307 (0.228)	0.026 (0.176)	0.277 (0.149)
Sample Size	**0.321 (0.096)**	**0.519 (0.105)**	**0.296 (0.149)**
PC1	−0.031 (0.115)	0.218 (0.123)	−0.261 (0.181)
PC2	−0.116 (0.095)	−0.002 (0.109)	**−0.332 (0.160)**
PC3	0.115 (0.101)	**0.354 (0.119)**	−0.261 (0.178)
PC4	0.025 (0.101)	0.076 (0.121)	0.009 (0.154)

Values with bold font had 95% confidence intervals that did not overlap 0

## Discussion

We found that current density was not a good predictor of multi-species genetic connectivity across our broad Ontario study area. In contrast, high current density was inversely related to gene flow in the southern, highly fragmented portion of our study. Across the full study area, our node-based measures of gene flow were more influenced by sample size than landscape structure. However, when we conducted separate analyses of northern and southern Ontario, we found that both cost and current density were important, depending on location, suggesting an effect of landscape context. In the northern analysis, natural cover was abundant and habitat fragmentation was not pronounced. Genetic connectivity of marten and lynx was associated with both sample effort and mean cost measured at various spatial scales. Higher cost was associated with reduced gene flow.

In contrast to northern Ontario, the southern portion of our study area was more heavily affected by development, urbanization, and habitat loss. Portions of this area had natural cover below the 30% threshold suggested by Andrén [53] as being associated with habitat fragmentation effects. In this region, we found that genetic connectivity was negatively associated with current density. In other words, within the context of a fragmented landscape, our current density map was inversely related to genetic connectivity or gene flow. Our results seem to provide further support for the hypothesis that that landscape pattern is important for some species only when the amount of suitable habitat is low [53–55]. For example, Betts et al. [56] found that the independent effects of habitat fragmentation were important for accurately modelling songbird occurrence, but only when habitat suitability in the landscape was low [56]. Our data suggest that high current density is associated with reduced gene flow when habitat amount is low.

**Fig. 3:**
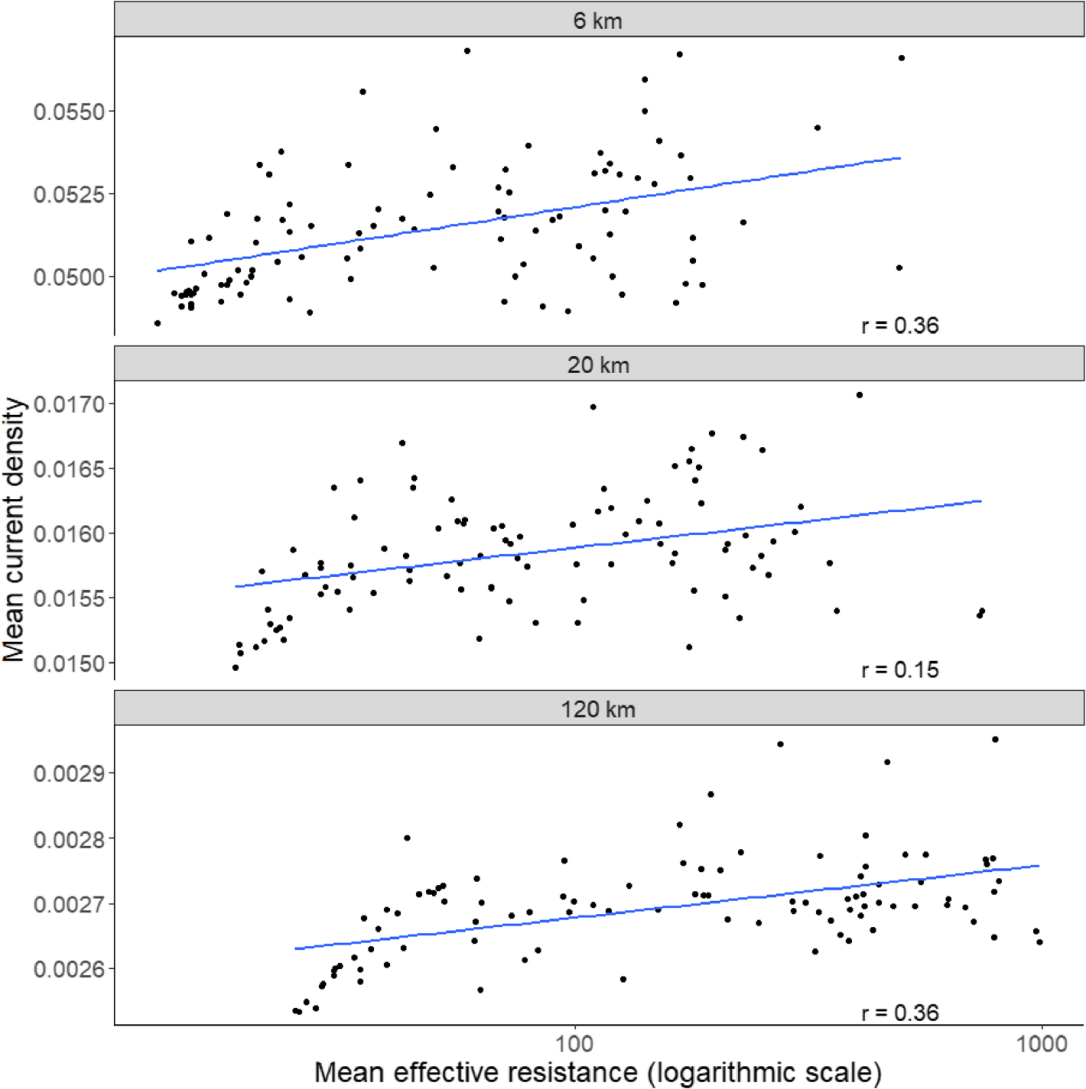
Comparison of current density and effective resistance in circular landscapes with radii of 6, 20, and 120 km. For each node where Canada lynx, American marten, fisher or southern flying squirrel were sampled we performed a pairwise Circuitscape analysis within the circular confines of all 3 neighborhood sizes. For each of these sampling nodes and neighborhoods, we placed 5 focal nodes equidistant around each site and calculated the effective resistance between the next-nearest neighbors. To further reduce the effect of the map boundary on the analysis we placed these focal nodes 300 m from the circular boundary of the landscape. We then calculated the mean effective resistance (ohms) between pairs and the mean current density across the circular landscape

We were surprised about the direction of the relationship between genetic connectivity and current density. We expected that high current density would be indicative of multi-species gene flow hot spots; however, we found that current density was negatively associated with genetic connectivity. To explore this further, we evaluated the relationship between point samples of current density and effective resistance estimated over the three neighbourhood sizes. Effective resistance is the cost of moving across a circuit between two points, and can be considered a pairwise measure of isolation [28]. We estimated effective resistance of each neighbourhood by connecting source and destination points around every neighbourhood’s perimeter. The relationship between current density and effective resistance was positive across all neighbourhood sizes, demonstrating that mean current density was higher in landscapes with high effective resistance (Fig. 3). In other words, landscapes with low habitat amount (and therefore high effective resistance) would be expected to have a high mean current density. While current density may be proportional to probability of movement, pinch points with high current density may only occur in proximity to areas with high resistance (i.e., low habitat amount). This point is underscored by the relationship between mean cost and mean current, which was negatively correlated in all but one of 9 comparisons (Additional file 1: Appendix 2), demonstrating that wide swaths of habitat have relatively low movement probability (i.e., by definition they are not pinch points). While these wide swaths of habitat may have low movement probability per unit area, they nevertheless appear to contribute to gene flow, likely as sources of suitable habitat for settlement and reproduction [35].

Perhaps not surprisingly, sample effort was the most important variable associated with genetic connectivity in all of our models. More samples at a site were associated with higher estimates of genetic connectivity (*r* = 0.25). We assume that this effect was a consequence of cGD being sensitive to under sampled sites [57]. This underscores the importance of sufficient sampling effort when carrying out studies of genetic connectivity. Insufficiently sampled sites may yield underestimates of allelic richness that result in biased estimated of genetic connectivity among sites, especially for frequency-based measures such as cGD. On the other hand, Koen et al. [57] showed that investigators should invest more in sampling additional sites, rather than sampling sites more intensively. A sample of at least 25-30 individuals may be required to accurately estimate allele frequencies however [58], and some of our nodes had fewer than this number of samples (Additional file 1: Appendix 3).

Local habitat fragmentation reduces genetic connectivity for multiple species, yet wildlife management efforts continue to rely heavily on single-species connectivity estimates to inform movement corridor planning. We believe such conservation strategies would benefit from multi-species connectivity estimates, as these estimates provide a simple and practical means to mitigate the effects of habitat fragmentation for a larger number of species. Our point-based connectivity analysis used circuit theory, with measures taken to avoid the spurious correlations commonly found when using pairwise measures of connectivity. Thus, our approach can estimate local movement probability, and might be used more reliably in some analyses than pairwise connectivity measures. Given the novelty of using circuit theory as a point-based estimator, we encourage further validation of this approach. With reliable, node-based estimates of landscape connectivity and multi-species genetic connectivity, wildlife managers can validate existing natural heritage plans as well as develop well-informed strategies that have the potential to benefit multiple species in a habitat.

There are limitations to modelling animal movement patterns across large landscapes using land cover resistance grids that led us to model connectivity over smaller areas to be compiled as tiles. For example, there are constraints on the number of pixels that can be processed by computing resources using Circuitscape, limiting the ability to run circuit models on rasters with large numbers of pixels representing vast landscape areas or even rasters representing fine scale habitats. Thus, resistance grids are often segmented into manageable units defined by artificial or natural map boundaries that are subsequently tiled together to create seamless resistance surfaces [44, 59]. Buffers incorporating surrounding land cover data around each tile are used to minimize border effects such as seam lines between tiles by creating an overlapping current density calculation area [11, 60]. Future large-scale connectivity analyses may benefit from coding and computer processing improvements that increase the area that can be mapped during processing runs, limiting the need for tiles (e.g., [61]).

Modelling multi-species connectivity has the potential to identify areas that are conducive to ecological flow, namely movement, dispersal and gene flow and such areas may be prioritized by natural heritage planners and conservation initiatives. Practitioners of multi-species connectivity modelling that aim to validate resistance surfaces by assessing the degree of gene flow between populations for a suite of species should be wary of habitat requirements and the associated movement ecology of each species as some species may insufficiently reflect landscape connectivity [62]. For example, carnivores have been thought to be an effective umbrella species to assess landscape connectivity due to their large home ranges [63] and capacity for long-distance dispersal [64]; however, it has been shown that such taxa are ineffective umbrella representatives for a majority of species due their specific habitat requirements that do not reflect the majority of species that are at risk of habitat degradation [10, 62]. The taxa we used in this study were selected based on their readily available population genetic data, and it is possible that these species are not reliable connectivity predictors of our provincial current density map. For example, Cushman and Landguth [25] showed that American marten was a poor indicator of landscape connectivity using resistant kernel modelling [10]. American marten are high elevation forest habitat specialists, and typically, species occupying lower elevations are more at risk of habitat degradation. This outlines the importance of selecting broadly applicable taxa when assessing resistance surfaces.

## Conclusion

We were able to demonstrate that multi-species landscape connectivity can be modelled but that effects may be dependent on landscape context. When habitat was abundant genetic connectivity was not related to current density, but instead had a positive relationship with habitat amount. Current density was more important in the southern part of our study area where habitat was less abundant. This finding shows the importance of landscape context, and appears to support the hypothesis that landscape pattern matters when habitat amount is low. Across all models, sample effort was important, suggesting that cGD is sensitive to undersampling.

## Additional file

Additional file 1: Supplementary material for Marrotte et al., Multi-species genetic connectivity in a terrestrial habitat network. (PDF 449 kb)

## Abbreviations

cGD: Conditional genetic distance
DNA: Deoxyribonucleic acid
LCP: Least cost path
PC: Principal component
