# Supplementary material for: Multi-species genetic connectivity in a terrestrial habitat network
Source: Mov Ecol. 2017 Oct 6;5:21. doi: 10.1186/s40462-017-0112-2 (PMC5629812; doi:10.1186/s40462-017-0112-2)
Supplement: Supplementary file 1 — Supplementary material for Marrotte et al., Multi-species genetic connectivity in a terrestrial habitat network. (PDF 449 kb) [file 40462_2017_112_MOESM1_ESM.pdf]

Additional file 1.

Supplementary material for Marrotte et al., Multi-species genetic connectivity in a terrestrial habitat network.

Appendix 1. Description of cost values for different land cover elements in a study of multi-species genetic connectivity in Ontario, Canada. Cost estimates followed the methodology of Koen et al. [11].

Data source	Publisher	Land cover	Resistance
[1]Built-up Area	OMNRF, 2013	Urban - (Population ≥ 1000)	1000
[2]Ontario Hydro Network (OHN), 500k Waterbody	OMNRF, 2011	Water Bodies (Area ≥ 100m2)	1000
[3]Ontario Road Network (ORN)	OMNRF, 2010	Arterial	1000
[3]Ontario Road Network (ORN)	OMNRF, 2010	Collector	100
[3]Ontario Road Network (ORN)	OMNRF, 2010	Expressway / Highway	1000
[3]Ontario Road Network (ORN)	OMNRF, 2010	Local / Strata	100
[3]Ontario Road Network (ORN)	OMNRF, 2010	Local / Street	100
[3]Ontario Road Network (ORN)	OMNRF, 2010	Local / Unknown	100
[3]Ontario Road Network (ORN)	OMNRF, 2010	Ramp	1000
[3]Ontario Road Network (ORN)	OMNRF, 2010	Resource / Recreation	100
[4]Southern Ontario Land Resource Information System v.1.2 (2000-2002)	OMNRF, 2007	Open Cliff and Talus	1000
[4]Southern Ontario Land Resource Information System v.1.2 (2000-2002)	OMNRF, 2007	Alvar	10
[4]Southern Ontario Land Resource Information System v.1.2 (2000-2002)	OMNRF, 2007	Shoreline	10
[4]Southern Ontario Land Resource Information System v.1.2 (2000-2002)	OMNRF, 2007	Open Shoreline	10
[4]Southern Ontario Land Resource Information System v.1.2 (2000-2002)	OMNRF, 2007	Open Sand Barren and Dune	10
[4]Southern Ontario Land Resource Information System v.1.2 (2000-2002)	OMNRF, 2007	Treed Sand Barren and Dune	10
[4]Southern Ontario Land Resource Information System v.1.2 (2000-2002)	OMNRF, 2007	Open Tallgrass Prairie	10
[4]Southern Ontario Land Resource Information System v.1.2 (2000-2002)	OMNRF, 2007	Tallgrass Savannah	10
[4]Southern Ontario Land Resource Information System v.1.2 (2000-2002)	OMNRF, 2007	Tallgrass Woodland	10
[4]Southern Ontario Land Resource Information System v.1.2 (2000-2002)	OMNRF, 2007	Forest	10
[4]Southern Ontario Land Resource Information System v.1.2 (2000-2002)	OMNRF, 2007	Coniferous Forest	10

[4]Southern Ontario Land Resource Information System v.1.2 (2000-2002)	OMNRF, 2007	Mixed Forest	10
[4]Southern Ontario Land Resource Information System v.1.2 (2000-2002)	OMNRF, 2007	Deciduous Forest	10
[4]Southern Ontario Land Resource Information System v.1.2 (2000-2002)	OMNRF, 2007	Plantations - Tree Cultivated	10
[4]Southern Ontario Land Resource Information System v.1.2 (2000-2002)	OMNRF, 2007	Hedge Rows	10
[4]Southern Ontario Land Resource Information System v.1.2 (2000-2002)	OMNRF, 2007	Transportation	1000
[4]Southern Ontario Land Resource Information System v.1.2 (2000-2002)	OMNRF, 2007	Extraction	1000
[4]Southern Ontario Land Resource Information System v.1.2 (2000-2002)	OMNRF, 2007	Built-up Area Previous	100
[4]Southern Ontario Land Resource Information System v.1.2 (2000-2002)	OMNRF, 2007	Built-up Area Impervious	1000
[4]Southern Ontario Land Resource Information System v.1.2 (2000-2002)	OMNRF, 2007	Swamp	10
[4]Southern Ontario Land Resource Information System v.1.2 (2000-2002)	OMNRF, 2007	Fen	10
[4]Southern Ontario Land Resource Information System v.1.2 (2000-2002)	OMNRF, 2007	Bog	10
[4]Southern Ontario Land Resource Information System v.1.2 (2000-2002)	OMNRF, 2007	Marsh	10
[4]Southern Ontario Land Resource Information System v.1.2 (2000-2002)	OMNRF, 2007	Open Water	1000
[5]Ontario Land Cover Data Base, Second Edition (2000)	Spectranalysis Inc., 2004	Water - deep clear	1000
[5]Ontario Land Cover Data Base, Second Edition (2000)	Spectranalysis Inc., 2004	Water - shallow / sedimented	1000
[5]Ontario Land Cover Data Base, Second Edition (2000)	Spectranalysis Inc., 2004	Settlement / Infrastructure	1000
[5]Ontario Land Cover Data Base, Second Edition (2000)	Spectranalysis Inc., 2004	Sand / Gravel / Mine Tailings	1000
[5]Ontario Land Cover Data Base, Second Edition (2000)	Spectranalysis Inc., 2004	Bedrock	10
[5]Ontario Land Cover Data Base, Second Edition (2000)	Spectranalysis Inc., 2004	Mudflats	10
[5]Ontario Land Cover Data Base, Second Edition (2000)	Spectranalysis Inc., 2004	Forest Depletion - cuts	10
[5]Ontario Land Cover Data Base, Second Edition (2000)	Spectranalysis Inc., 2004	Forest Depletion - burns	10
[5]Ontario Land Cover Data Base, Second Edition (2000)	Spectranalysis Inc., 2004	Forest - regenerating depletion	10
[5]Ontario Land Cover Data Base, Second Edition (2000)	Spectranalysis Inc., 2004	Forest - sparse	10
[5]Ontario Land Cover Data Base, Second Edition (2000)	Spectranalysis Inc., 2004	Forest - dense deciduous	10
[5]Ontario Land Cover Data Base, Second Edition (2000)	Spectranalysis Inc., 2004	Forest - dense mixed	10
[5]Ontario Land Cover Data Base, Second Edition (2000)	Spectranalysis Inc., 2004	Forest - dense coniferous	10
[5]Ontario Land Cover Data Base, Second Edition (2000)	Spectranalysis Inc., 2004	Marsh - intertidal	10
[5]Ontario Land Cover Data Base, Second Edition (2000)	Spectranalysis Inc., 2004	Marsh - supertidal	10
[5]Ontario Land Cover Data Base, Second Edition (2000)	Spectranalysis Inc., 2004	Marsh - inland	10

[5]Ontario Land Cover Data Base, Second Edition (2000)	Spectranalysis Inc., 2004	Swamp - deciduous	10
[5]Ontario Land Cover Data Base, Second Edition (2000)	Spectranalysis Inc., 2004	Swamp - coniferous	10
[5]Ontario Land Cover Data Base, Second Edition (2000)	Spectranalysis Inc., 2004	Fen - open	10
[5]Ontario Land Cover Data Base, Second Edition (2000)	Spectranalysis Inc., 2004	Fen - treed	10
[5]Ontario Land Cover Data Base, Second Edition (2000)	Spectranalysis Inc., 2004	Bog - Open	10
[5]Ontario Land Cover Data Base, Second Edition (2000)	Spectranalysis Inc., 2004	Bog - treed	10
[5]Ontario Land Cover Data Base, Second Edition (2000)	Spectranalysis Inc., 2004	Tundra Heath	10
[5]Ontario Land Cover Data Base, Second Edition (2000)	Spectranalysis Inc., 2004	Agriculture - Pasture / abandoned fields	100
[5]Ontario Land Cover Data Base, Second Edition (2000)	Spectranalysis Inc., 2004	Agriculture - cropland	100
[6]Ontario Land Cover Data Base, First Edition (1998)	OMNRF, 1998	Water	1000
[6]Ontario Land Cover Data Base, First Edition (1998)	OMNRF, 1998	Coastal Mudflats	10
[6]Ontario Land Cover Data Base, First Edition (1998)	OMNRF, 1998	Intertidal Marsh	10
[6]Ontario Land Cover Data Base, First Edition (1998)	OMNRF, 1998	Supertidal Marsh	10
[6]Ontario Land Cover Data Base, First Edition (1998)	OMNRF, 1998	Freshwater Coastal Marsh / Inland Marsh	10
[6]Ontario Land Cover Data Base, First Edition (1998)	OMNRF, 1998	Deciduous Swamp	10
[6]Ontario Land Cover Data Base, First Edition (1998)	OMNRF, 1998	Conifer Swamp	10
[6]Ontario Land Cover Data Base, First Edition (1998)	OMNRF, 1998	Open Fen	10
[6]Ontario Land Cover Data Base, First Edition (1998)	OMNRF, 1998	Treed Fen	10
[6]Ontario Land Cover Data Base, First Edition (1998)	OMNRF, 1998	Open Bog	10
[6]Ontario Land Cover Data Base, First Edition (1998)	OMNRF, 1998	Treed Bog	10
[6]Ontario Land Cover Data Base, First Edition (1998)	OMNRF, 1998	Tundra Heath	10
[6]Ontario Land Cover Data Base, First Edition (1998)	OMNRF, 1998	Dense Deciduous Forest	10
[6]Ontario Land Cover Data Base, First Edition (1998)	OMNRF, 1998	Dense Coniferous Forest	10
[6]Ontario Land Cover Data Base, First Edition (1998)	OMNRF, 1998	Coniferous Plantation	10
[6]Ontario Land Cover Data Base, First Edition (1998)	OMNRF, 1998	Mixed Forest – mainly Deciduous	10
[6]Ontario Land Cover Data Base, First Edition (1998)	OMNRF, 1998	Mixed Forest – mainly Coniferous	10
[6]Ontario Land Cover Data Base, First Edition (1998)	OMNRF, 1998	Sparse Coniferous Forest	10
[6]Ontario Land Cover Data Base, First Edition (1998)	OMNRF, 1998	Sparse Deciduous Forest	10
[6]Ontario Land Cover Data Base, First Edition (1998)	OMNRF, 1998	Recent Cutovers (now >10yrs of age)	10

[6]Ontario Land Cover Data Base, First Edition (1998)	OMNRF, 1998	Old Cuts and Burns (>10yrs of age)	10
[6]Ontario Land Cover Data Base, First Edition (1998)	OMNRF, 1998	Bedrock Outcrop	10
[6]Ontario Land Cover Data Base, First Edition (1998)	OMNRF, 1998	Settlement and Developed Land	1000
[6]Ontario Land Cover Data Base, First Edition (1998)	OMNRF, 1998	Pasture and Abandoned Fields	100
[6]Ontario Land Cover Data Base, First Edition (1998)	OMNRF, 1998	Cropland	100
[6]Ontario Land Cover Data Base, First Edition (1998)	OMNRF, 1998	Alvar	10

*Superscript numbers represent cost surface development overlay priority. Priority 3 land cover data was only included in small undifferentiated areas.

Appendix 2. Correlation matrix for all variables considered in a multi-species genetic connectivity study in Ontario, Canada.

	Sample Size	X	Y	Σ of Edge Wt	Σ of Inv Edge Wt	# of Edges	Avg Edge Wt	Avg Inv Edge Wt	Mean Cost (6 km)	Mean Cost (20 km)	Mean Cost (120 km)	Mean Current (6 km)	Mean Current (20 km)	Mean Current (120 km)	STD Current (6 km)	STD Current (20 km)	STD Current (120 km)	STD Cost (6 km)	STD Cost (20 km)
Sample Size																			
X	-0.055																		
Y	0.049	-0.837																	
Σ of Edge Wt	-0.254	0.231	-0.352																
Σ of Inv Edge Wt	0.006	-0.476	0.563	0.005															
# of Edges	-0.196	-0.133	0.096	0.719	0.677														
Avg Edge Wt	-0.239	0.498	-0.648	0.810	-0.542	0.213													
Avg Inv Edge Wt	0.245	-0.571	0.721	-0.696	0.608	-0.137	-0.944												
Mean Cost (6 km)	0.186	0.222	-0.400	0.055	-0.326	-0.197	0.237	-0.238											

Mean Cost (20 km)	0.105	0.178	-0.358	0.064	-0.240	-0.124	0.220	-0.228	0.627										
Mean Cost (120 km)	0.003	0.037	-0.344	0.118	-0.352	-0.134	0.330	-0.383	0.368	0.560									
Mean Current (6 km)	-0.136	-0.001	-0.057	0.163	0.034	0.130	0.166	-0.137	-0.429	-0.224	-0.040								
Mean Current (20 km)	-0.065	-0.007	-0.121	0.280	-0.101	0.112	0.321	-0.275	-0.122	-0.263	-0.012	0.772							
Mean Current (120 km)	-0.015	-0.021	-0.180	0.327	-0.123	0.119	0.342	-0.274	0.042	-0.005	-0.026	0.453	0.686						
STD Current (6 km)	-0.098	0.113	-0.189	0.115	-0.198	-0.086	0.235	-0.170	0.316	0.262	0.020	0.270	0.333	0.319					
STD Current (20 km)	-0.047	0.031	-0.097	0.097	-0.115	-0.043	0.173	-0.110	0.210	0.435	0.040	0.364	0.421	0.411	0.700				
STD Current (120 km)	-0.038	-0.116	0.040	0.106	-0.125	-0.043	0.155	-0.089	0.007	0.101	0.109	0.322	0.518	0.731	0.365	0.604			
STD Cost (6 km)	0.108	0.194	-0.369	0.146	-0.283	-0.100	0.279	-0.270	0.807	0.566	0.343	-0.315	-0.049	0.092	0.592	0.293	0.050		
STD Cost (20 km)	0.099	0.143	-0.309	0.071	-0.208	-0.092	0.192	-0.204	0.563	0.956	0.546	-0.195	-0.248	-0.005	0.296	0.444	0.096	0.614	
STD Cost (120 km)	-0.002	-0.002	-0.289	0.082	-0.304	-0.125	0.266	-0.321	0.339	0.584	0.963	-0.034	-0.025	-0.025	0.025	0.060	0.113	0.366	0.616

Appendix 3. Description of data from each location sampled for four different species in a multi-species genetic connectivity study in Ontario, Canada.

Species	Node ID	X	Y	Sample Size	Avg Edge Wt	Avg Inv Edge Wt	Mean Cost			Mean Current Density		
							6km Buffer	20 km Buffer	120 km Buffer	6 km Buffer	20 km Buffer	120 km Buffer
Fisher	F1	1739622	12011268	20	10.130	0.100	NA	NA	NA	NA	NA	NA
	F2	1445630	12133088	20	4.138	0.245	122.4	163.8	155.9	1.579	0.317	0.255
	F3	1533340	12050250	15	7.189	0.141	55.9	126.1	133.6	0.789	0.405	0.046
	F4	1419389	12051416	24	4.238	0.243	264.3	175.7	210.8	0.075	0.222	-0.013
	F5	1467476	12046021	23	4.505	0.227	137.8	151.6	168.2	0.573	0.156	0.032
	F6	1303506	12213490	20	4.459	0.231	114.2	111.7	215.9	-0.187	-0.335	-0.127
	F7	1497090	12013919	6	8.987	0.114	386.2	155.9	157.8	-0.344	-0.118	-0.060
	F8	1286463	12156737	26	5.746	0.180	17.3	83.3	175.5	0.279	0.219	0.095
	F9	1561398	12105898	16	4.965	0.202	142.7	163.1	145.0	-0.105	-0.317	-0.197
	F10	1313131	12133971	15	4.210	0.239	378.8	262.1	172.7	-0.574	-0.020	0.045
	F11	1309879	12106002	6	6.477	0.169	176.4	213.4	195.7	0.088	-0.203	0.055
	F12	1500689	12102156	19	5.007	0.204	65.1	125.3	138.5	-0.108	-0.197	0.021
	F13	1332743	12072990	14	8.687	0.118	277.1	285.8	223.2	0.191	0.363	-0.024
	F14	1595910	12080118	18	4.689	0.216	138.0	115.6	172.6	-0.371	-0.224	-0.091
	F15	1594663	12102987	19	4.967	0.207	58.3	116.4	164.1	0.380	-0.021	-0.189
	F16	1214171	12059184	25	8.096	0.127	183.2	81.5	115.3	0.991	1.697	1.276
	F17	1650798	12033546	19	5.914	0.183	91.2	205.3	204.5	0.211	0.017	0.066
	F18	1296197	12181004	19	4.095	0.249	143.8	168.3	185.5	-0.445	-0.096	0.005
	F19	1513164	12149559	19	6.098	0.173	116.3	181.0	129.8	0.014	-0.194	-0.124
	F20	1442571	12035753	20	4.112	0.247	131.2	99.5	180.2	0.222	0.303	-0.002
	F21	1634857	12158264	18	6.361	0.168	NA	NA	NA	NA	NA	NA
	F22	1540424	12011498	13	7.223	0.143	148.0	113.8	140.0	-0.331	-0.169	-0.052
	F23	1563908	12044767	23	5.343	0.193	301.4	121.6	157.0	-0.187	0.351	-0.020
	F24	1597829	12028458	29	5.215	0.196	351.1	299.7	189.6	0.027	0.035	-0.066
	F25	1528965	12104651	17	5.876	0.176	66.2	91.0	141.6	-0.157	-0.155	-0.098
	F26	1506108	12028673	26	5.977	0.171	93.5	62.8	147.3	0.408	0.357	-0.020

	F27	1593416	12127105	22	4.689	0.215	160.0	169.1	154.6	-0.472	-0.568	-0.288
	F28	1641234	12081781	18	4.535	0.223	70.4	90.9	186.4	0.585	0.377	-0.063
	F29	1348173	12111161	22	4.755	0.219	76.9	114.5	200.8	0.276	0.197	-0.024
	F30	1378827	12220481	14	6.896	0.152	79.2	72.9	182.3	1.139	1.156	0.169
	F31	1376598	12024262	15	4.594	0.219	809.9	342.1	251.2	-1.080	-0.315	-0.027
	F32	1673840	12066493	44	6.141	0.177	99.3	99.0	170.1	0.548	0.511	0.101
	F33	1623354	12110888	20	4.301	0.233	129.0	139.2	179.0	-0.214	-0.297	-0.147
	F34	1568051	12147895	18	4.997	0.201	119.8	152.6	146.5	-0.592	-0.396	-0.344
Canada lynx	L1	1080606	12230197	18	4.290	0.233	194.8	156.6	156.5	-0.032	-0.088	-0.121
	L2	1270773	12562168	23	4.197	0.243	242.7	159.6	76.5	-1.124	-0.648	-0.238
	L3	1186138	12560719	27	3.431	0.294	42.9	86.5	71.2	0.519	0.352	-0.087
	L4	1087993	12410014	23	3.555	0.283	81.2	86.5	92.2	-0.145	-0.282	-0.164
	L5	1083299	12328081	22	3.872	0.264	79.9	98.8	120.2	-0.104	-0.021	-0.044
	L6	353239	12613973	24	4.598	0.224	48.1	87.1	241.4	0.108	0.045	0.109
	L7	825677	12610039	35	3.723	0.272	98.4	110.6	127.9	0.134	0.257	0.028
	L8	1190125	12331202	15	4.448	0.231	37.6	94.4	135.7	0.884	0.638	0.076
	L9	985797	12603072	25	3.843	0.261	16.5	38.6	45.9	0.132	0.068	0.055
	L10	983215	12507143	27	3.594	0.279	34.1	31.8	87.4	0.562	0.648	-0.078
	L11	481908	12581819	35	3.847	0.265	68.0	129.8	216.8	0.141	0.161	-0.015
	L12	1122231	12583756	20	3.629	0.276	73.9	61.7	57.7	0.098	0.256	0.082
	L13	1071151	12583920	26	3.839	0.268	50.7	28.4	49.7	0.048	0.107	0.124
	L14	1141818	12493607	32	3.115	0.323	14.3	33.1	47.3	-0.448	-0.371	0.069
	L15	1086098	12483816	22	4.053	0.251	36.2	32.5	46.8	-0.074	-0.159	-0.064
	L16	1285989	12421624	34	4.021	0.253	17.0	58.6	129.1	0.467	0.399	-0.122
	L17	1277264	12358912	29	3.970	0.253	150.8	88.1	119.2	-0.477	-0.305	-0.179
	L18	1345953	12211220	26	4.031	0.249	43.0	278.3	190.4	-0.401	-0.663	-0.002
	L19	717517	12610416	13	4.347	0.235	62.4	50.5	325.9	0.213	0.185	-0.326
	L20	320617	12775926	22	5.345	0.188	64.9	175.9	197.1	-0.240	-0.005	-0.002
	L21	472510	12677654	13	5.512	0.182	72.1	126.1	192.1	0.282	0.025	0.078
	L22	1003818	12251441	18	3.808	0.264	36.5	43.1	107.8	0.816	0.684	0.249
	L23	1226981	12238141	27	3.771	0.272	32.1	78.5	160.4	-0.377	-0.425	0.000
	L24	567015	12537094	30	3.716	0.270	171.6	185.0	161.7	0.143	0.052	0.051

	L25	578319	12445973	15	4.464	0.228	57.6	59.8	149.2	0.686	0.671	0.082
	L26	1196149	12426963	39	3.539	0.288	36.7	149.1	93.4	0.874	0.407	0.247
	L27	833475	12500732	27	3.384	0.297	268.2	47.2	102.7	-0.687	-0.240	-0.138
	L28	942374	12402154	35	3.282	0.305	201.5	144.4	136.8	-0.317	-0.163	0.058
Marten	M1	645709	12504228	27	3.325	0.302	88.7	135.1	177.3	0.465	0.412	0.164
	M2	307903	12765030	47	2.814	0.356	112.4	194.2	211.7	-0.138	-0.221	-0.018
	M3	331965	12746408	22	2.942	0.343	283.3	166.1	203.1	-0.061	0.044	0.000
	M4	1310577	12336637	23	3.579	0.287	22.4	93.9	147.1	0.276	0.053	-0.207
	M5	1314998	12299047	23	4.480	0.225	130.8	208.7	189.4	-0.427	-0.350	-0.074
	M6	772061	12564261	19	3.357	0.305	78.4	156.5	155.1	-0.033	0.099	-0.057
	M7	799255	12559363	20	3.068	0.330	28.3	282.4	149.8	1.123	-0.190	-0.073
	M8	790744	12472796	23	3.430	0.298	277.9	134.6	107.5	-0.472	0.127	-0.016
	M9	870372	12479829	22	3.499	0.287	18.2	77.4	103.3	-0.312	-0.305	-0.153
	M10	834988	12507045	22	3.559	0.281	27.5	79.3	104.7	-0.232	-0.405	-0.133
	M11	911795	12523140	23	3.175	0.318	52.4	69.2	92.8	0.191	-0.038	-0.192
	M12	930971	12563164	24	3.062	0.328	13.5	24.9	62.9	0.116	0.043	-0.055
	M13	995659	12504126	22	3.608	0.279	40.8	72.3	80.9	0.630	0.324	-0.091
	M14	1093430	12298965	23	3.935	0.261	122.5	118.2	136.4	-0.276	-0.137	-0.053
	M15	1087158	12306606	22	3.775	0.272	107.5	143.9	131.9	-0.162	-0.144	-0.045
	M16	1243724	12497029	25	3.509	0.303	24.6	45.3	100.0	0.286	0.155	-0.038
	M17	1254122	12480710	21	3.514	0.289	82.4	94.2	115.5	-0.351	-0.195	-0.057
	M18	1195369	12520878	27	3.400	0.300	54.8	77.6	64.7	1.018	0.567	0.049
	M19	1288274	12348563	16	3.745	0.271	276.8	86.2	129.8	-0.403	-0.090	-0.202
	M20	1330473	12368285	15	4.889	0.209	31.8	76.7	119.6	0.180	0.030	-0.244
	M21	781645	12493991	25	3.533	0.285	133.6	110.6	125.9	0.524	0.359	-0.047
	M22	1032093	12342170	16	4.237	0.238	45.1	104.6	116.5	0.704	0.106	-0.016
	M23	1018022	12480744	21	3.323	0.309	111.4	63.4	77.9	-0.376	-0.311	-0.088
	M24	768944	12541960	22	3.422	0.311	125.9	230.7	149.7	-0.716	-0.350	-0.065
	M25	913677	12537056	24	3.615	0.281	51.0	72.2	85.4	-0.023	-0.067	-0.167
	M26	1307631	12314745	26	3.904	0.259	88.8	211.0	175.6	-0.033	-0.128	-0.164
	M27	1169197	12362830	11	4.602	0.220	144.5	112.5	124.3	0.015	0.209	0.078
	M28	1222471	12478785	19	3.401	0.297	41.2	62.4	84.2	0.189	0.165	0.112

	M29	1436604	12125411	23	5.276	0.191	112.9	168.8	170.6	0.373	0.441	0.207
Flying Squirrel	S1	1428556	12124870	22	4.976	0.208	312.9	211.6	173.3	-0.023	0.235	0.194
	S2	1406621	12173163	6	6.037	0.169	257.9	170.9	146.8	-0.194	0.182	0.329
	S3	1413325	12081272	33	5.242	0.204	274.8	229.9	184.8	0.528	0.210	0.075
	S4	1458022	12030443	60	5.458	0.192	246.8	173.5	181.9	0.180	0.121	-0.047
	S5	1396129	12216839	11	5.252	0.200	81.4	102.1	162.0	0.836	0.713	0.326
	S6	1446435	12017165	120	4.140	0.246	525.4	243.7	188.5	-0.818	-0.193	-0.034
	S7	1208537	11773102	11	7.320	0.137	146.3	134.3	163.7	0.849	0.701	0.327
	S8	1413325	12081272	15	4.914	0.215	274.8	229.9	184.8	0.528	0.210	0.075
